# Effects of Ambulatory Blood Pressure Monitoring During Polysomnography on Sleep Indices and EEG Power

**DOI:** 10.3390/jcm15218759

**Published:** 2026-11-09

**Authors:** Jeonghoon Kim, Donghun Kang, Jongshill Lee, Junhee Han, Jeyeon Lee, Myung-Kyu Yi, Byung Sik Kim, Hyun-Jin Kim, Jinseok Park, Jeong-Hun Shin, In Young Kim

**Affiliations:** 1 Department of Electronic Engineering, Hanyang University, Seoul 04763, Republic of Korea; kjhoon6075@hanyang.ac.kr; 2 Department of Biomedical Engineering, Hanyang University, Seoul 04763, Republic of Korea; donghun2296@hanyang.ac.kr (D.K.); netlee@hanyang.ac.kr (J.L.); hanjh2000@hanyang.ac.kr (J.H.); kainos14@hanyang.ac.kr (M.-K.Y.); 3 Department of Biomedical Engineering, College of Medicine, Hanyang University, Seoul 04763, Republic of Korea; jeyeonlee@hanyang.ac.kr; 4 Division of Cardiology, Department of Internal Medicine, College of Medicine, Hanyang University Guri Hospital, Guri 11923, Republic of Korea; fish3777@hanyang.ac.kr (B.S.K.); titi8th@hanyang.ac.kr (H.-J.K.); cardio.hyapex@gmail.com (J.-H.S.); 5 Department of Neurology, College of Medicine, Hanyang University Seoul Hospital, Seoul 04763, Republic of Korea; jinspark@hanyang.ac.kr

**Keywords:** ambulatory blood pressure monitoring, polysomnography, first-night effect, sleep architecture, electroencephalography, arousal, crossover study

## Abstract

**Background/Objectives:** Ambulatory blood pressure monitoring (ABPM) may disturb sleep during polysomnography (PSG), and the first-night effect (FNE) can complicate interpretation. We evaluated changes associated with ABPM and FNE across whole nights and individual measurements. **Methods:** This secondary analysis included 72 nominally healthy adults in a balanced two-night crossover, with ABPM on one randomly assigned night. Linear mixed-effects models estimated associations with PSG-derived sleep indices, a composite sleep quality index (SQI), and relative electroencephalographic (EEG) power. Analyses of individual measurements summarized EEG changes, sleep-stage transitions, and automated arousal detections. Benjamini–Hochberg false discovery rate (BH-FDR) adjustment was applied within defined analysis families. **Results:** FNE and ABPM were associated with lower SQI (β = −0.049 and −0.051, respectively; both BH-adjusted *p* = 0.028). FNE was associated with altered sleep continuity and architecture, whereas ABPM was associated with higher light-sleep and lower N3-sleep percentages and higher whole-night relative theta power. In the Night 2 paired analysis, relative delta power was lower and relative alpha, beta, and gamma power were higher during ABPM than before ABPM (all BH-adjusted *p* < 0.01); the theta difference did not reach significance (BH-adjusted *p* = 0.0632). Of 655 eligible ABPM measurements beginning during sleep, 395 (60.3%) contained at least one arousal detection within the 2 min measurement window. **Conclusions:** These detections indicate temporal co-occurrence and do not establish that cuff inflation caused arousal. Concurrent ABPM and study-night order should be considered when interpreting PSG. An adaptation PSG night may be considered when feasible, as may cuffless blood pressure monitoring once its accuracy has been established for the intended setting.

## 1. Introduction

Polysomnography (PSG) is the reference method for assessing sleep stages, sleep continuity, respiratory events, movement, and neurophysiological activity. The laboratory environment and recording procedures can influence sleep. An unfamiliar room, attached sensors, and observation may cause sleep on the first recording night to differ from that on subsequent nights, a phenomenon known as the first-night effect (FNE) [1]. Studies in healthy adults have reported lower sleep efficiency and greater wakefulness on the first night [2]. Neurophysiological findings also suggest that vigilance to external stimuli may persist in an unfamiliar sleep environment [3]. FNE may therefore confound the interpretation of an experimental condition assigned to the first night.

Ambulatory blood pressure monitoring (ABPM) is widely used to measure blood pressure patterns during daily life and sleep. Repeated nocturnal cuff inflation and device noise may, however, disturb sleep. Early PSG studies found that cuff measurements often coincided with electroencephalographic (EEG) arousal [4] and were associated with less slow-wave sleep, longer arousals, or lower sleep efficiency [5]. Small experimental studies also reported greater wakefulness or transient EEG arousal after cuff inflation [6]. Other studies found little objective evidence of sleep disruption: subjective discomfort did not consistently correspond to objective sleep measures [7], actigraphic changes in healthy adults were small [8], and a recent randomized two-night crossover study found minimal changes in major PSG outcomes [9]. Differences in sample characteristics, monitoring intervals, equipment, sleep-assessment methods, and control of FNE may contribute to these inconsistent findings.

Many earlier studies did not account for FNE or ABPM sequence and examined either whole-night outcomes or short-term responses to individual cuff measurements [4,5,6,7,8,9]. Whole-night analyses capture changes in sleep architecture and continuity but may dilute transient responses. Analyses of individual measurements characterize EEG and arousal responses around cuff measurements but do not describe overall changes across the night; repeated measurements are also clustered within participants. To examine both, we used a balanced two-night PSG crossover in which ABPM was assigned to Night 1 or Night 2. A linear mixed-effects model (LMM) estimated ABPM and FNEs jointly while accounting for ABPM sequence and repeated observations. We combined whole-night analyses of sleep indices and relative EEG band power with analyses of EEG changes, sleep-stage transitions, and arousal occurrence around individual cuff measurements.

The primary aims were to (1) estimate night-level ABPM and FNEs on sleep indices and relative EEG band power using an LMM that accounted for ABPM sequence and repeated observations, and (2) characterize cuff-related changes in relative EEG band power at the participant level and sleep-stage transitions and arousal occurrence at the measurement level. Exploratory analyses examined these associations separately in participants with relatively higher and lower sleep quality.

## 2. Materials and Methods

### 2.1. Measurement Protocol

The Centre for Sleep and Cognition, National University of Singapore, collected and provided the data used in this study [10,11]. The recordings analyzed here were collected approximately between June 2021 and February 2022. The parent study was approved by the Institutional Review Board of the National University of Singapore and conducted in accordance with the Declaration of Helsinki. All participants provided written informed consent [10].

#### 2.1.1. Participants

Nominally healthy adults were recruited for the parent study. The participant information sheet specified the following eligibility criteria: age 21–70 years, body mass index (BMI) 18–35 kg/m^2^, no diagnosed sleep, neurological, or psychiatric disorders, an Epworth Sleepiness Scale score ≤ 10, and no high risk of obstructive sleep apnea on the Berlin questionnaire. Additional requirements were habitual sleep of at least 5 h/night within 20:00–10:00, no pregnancy or acute illness, no use of wake-promoting agents, stimulants, or sodium oxybate, and the ability to move without assistance. Participants attended a daytime briefing and two overnight PSG sessions. ABPM was performed during one PSG night and omitted during the other, with the order randomized. Participants arrived approximately 2 h before their habitual bedtime and underwent a 15 min seated baseline recording after equipment setup. Lights-off and lights-on times followed each participant’s habitual sleep and wake times [10]. The parent-study protocol specified a minimum of two overnight sessions and permitted an intervening night when required. Demographic and baseline characteristics of the participants, overall and by randomized ABPM sequence, are summarized in Table 1.

#### 2.1.2. PSG Measurement

Height, weight, waist circumference, and office blood pressure were measured at the briefing. PSG was recorded using a SOMNOtouch system (SOMNOmedics GmbH, Randersacker, Germany). EEG was recorded from C3 and C4, referenced to the contralateral mastoids (C3–A2 and C4–A1); Fpz and Cz served as the ground and recording reference, respectively. Bilateral electrooculography (EOG), submental electromyography (EMG), thoracic respiratory effort, electrocardiography (ECG), and fingertip photoplethysmography (PPG) were recorded concurrently [10,11]. EEG, EOG, EMG, ECG, and PPG were sampled at 256 Hz, and thoracic respiratory effort at 32 Hz. In this analysis, EEG was used for spectral analysis and arousal detection, and submental EMG was used to apply the REM-specific arousal criterion.

#### 2.1.3. Sleep-Stage Annotations and Arousal Detection

This secondary analysis used sleep-stage annotations provided by the original research team. As reported by Yilmaz et al. [10], the annotations combined outputs from Neurobit PSG (Neurobit Inc., New York, NY, USA), Somnolyzer 24 × 7 (The Siesta Group Schlafanalyse GmbH, Vienna, Austria), and the U-Sleep web service (University of Copenhagen, Copenhagen, Denmark). Software information is available at https://www.neurobit.com/publications/psg, https://thesiestagroup.com/solutions/central-scoring/, and https://sleep.ai.ku.dk/, respectively (accessed on 26 September 2026). Trained in-house staff checked the Neurobit output, and Siesta Group experts reviewed the Somnolyzer output. Labels for 30 s epochs were assigned by majority vote; when all three outputs differed, the Neurobit label was retained. Validation studies of the individual systems have been published elsewhere [12,13,14]. The source study reported 30 s sleep-stage and wake after sleep onset (WASO) assessments according to AASM criteria, citing the first-edition manual [10,15]. We did not rescore the supplied annotations according to AASM Version 3 (2023) [16].

Arousal-related events were identified separately from the supplied sleep-stage annotations using a study-specific automated detector developed with reference to AASM Version 2.0 (2012) and the official February 2023 summary of updates in Version 3 [17,18]. Selected requirements for event duration, preceding sleep, spindle exclusion, and REM-associated chin EMG activity were implemented using research-defined numerical thresholds and signal-processing procedures. Throughout this study, arousal detections refer to events identified by this detector.

The C3–A2 and C4–A1 waveforms were averaged before detection. After estimated spindle intervals were removed, band-power ratios were used to identify candidate segments lasting at least 3 s. Preceding sleep was assessed over 10 s using the supplied sleep-stage labels and detection history. Candidates with REM as their modal stage required chin EMG elevation to at least twice the preceding 10 s baseline median for at least 1 s, overlapping the candidate and REM labels. The same algorithm and fixed settings were applied to all participants across the four combinations of study night (Night 1 or Night 2) and ABPM status (present or absent). Detection criteria, signal-processing parameters, and event quantification are described in Supplementary Section S7 and Table S6.

#### 2.1.4. ABPM Measurements

ABPM measurements were obtained at 30 min intervals using a validated OnTrak 90227 monitor (Spacelabs Healthcare, Snoqualmie, WA, USA) [19]. The cuff was placed on the arm opposite the PPG sensor [11]. Participants with fewer than 10 ABPM measurements were excluded from the night-level analyses.

### 2.2. Sleep Indices and Whole-Night Relative EEG Band Power

Sleep indices and relative EEG band power were calculated for each participant-night. Analyses requiring a single value per participant used the mean across the two nights, whereas condition-specific analyses used the corresponding night-level value.

#### 2.2.1. Calculation of Sleep Indices

The durations of light sleep (N1 and N2 combined), N3 sleep, and REM sleep were expressed as percentages of total sleep time (TST) [15,20]. For arousal detections beginning during sleep, we calculated the total arousal count, arousal index, and arousal duration per hour. The arousal index was the number of events per hour of TST; arousal duration per hour was their cumulative duration, expressed in seconds per hour of TST. Event grouping and duration calculations are summarized in Supplementary Section S7 and Table S6.

#### 2.2.2. Construction and Application of the Composite Sleep Quality Index

SQI was constructed as a research composite that summarizes sleep duration, continuity, architecture, and fragmentation relative to the study sample. It included TST, sleep efficiency (SE), sleep-onset latency (SOL), percentages of N1, N3, and REM sleep, and arousal index. Components were selected to represent these domains while keeping all variance inflation factors (VIFs) ≤ 10 to limit excessive linear redundancy [21,22]. Sensitivity to component selection was examined across alternative component sets, with correlations and effect estimates summarized by maximum VIF (Supplementary Section S5; Figure S1 and Table S4).

For each component, observed values were ranked in the direction of better sleep and converted to relative scores, with 1 representing the best observed value. Higher TST, SE, N3-sleep percentage, and REM-sleep percentage, and lower SOL, N1-sleep percentage, and arousal index received higher scores. Rank scoring placed indices with different units on a common relative scale. The night-level SQI was the equally weighted mean of these component scores. Sensitivity analyses and correlations with individual sleep indices are summarized in Supplementary Sections S5 and S6.

**Figure 1:**
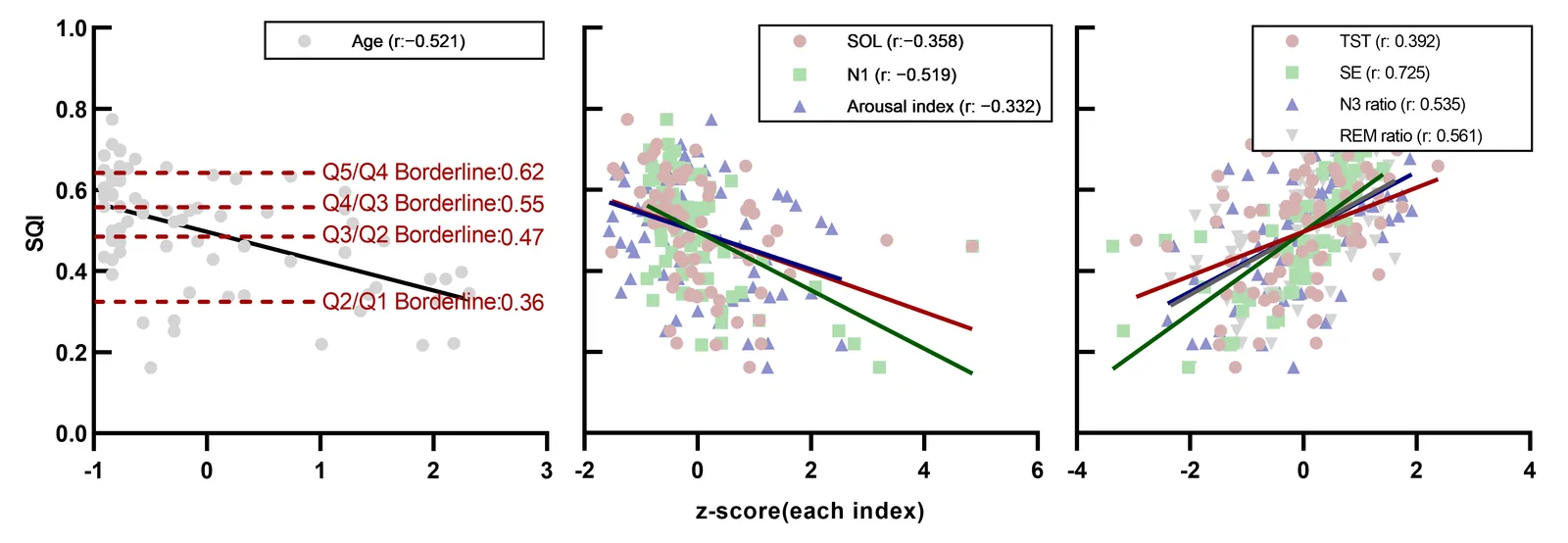
Associations of SQI with age and selected sleep indices. Each point represents one participant (*n* = 72). SQI and sleep indices were averaged across the two nights; age and sleep indices are expressed as *z* scores. Solid lines indicate linear fits, and the legends report Pearson correlation coefficients. Dashed horizontal lines mark the SQI quintile boundaries (0.36, 0.47, 0.55, and 0.62).

Participants were grouped by quintiles of their two-night mean SQI: Q1–Q2 formed the lower-SQI group and Q4–Q5 the higher-SQI group. Q3 was excluded only from SQI-stratified analyses. The quintile boundaries are shown in Figure 1. Separate LMMs with the same model structure estimated associations within each group while accounting for the other night-level effect, ABPM sequence, and participant-specific baseline differences. These analyses were exploratory and used unadjusted *p*-values.

#### 2.2.3. Calculation of Whole-Night Relative EEG Band Power

Relative EEG band power was calculated by estimating the power spectral density (PSD) and integrating it within each frequency band [23,24,25]. PSD was estimated separately for the C3–A2 and C4–A1 derivations using 30 s analysis windows advanced in 10 s steps. Within each window, Welch’s method used 4 s Hamming-windowed segments with 50% overlap, yielding a frequency resolution of 0.25 Hz [23,24]. Power was calculated for the delta (0.5–4 Hz), theta (4–8 Hz), alpha (8–13 Hz), beta (13–30 Hz), and gamma (30–45 Hz) bands. Relative power was the proportion of total power within 0.5–45 Hz contributed by each band [25]. Estimates from the two EEG derivations were averaged within each analysis window [26,27]. The whole-night value for each band was the median of all valid relative-power estimates obtained during sleep for that participant-night [28,29].

### 2.3. Linear Mixed-Effects Model Analysis and Estimation of Adjusted Values

We compared PSG-derived sleep indices and relative EEG band power between nights with and without ABPM. In the two randomized sequences, ABPM was performed on Night 1 or Night 2, so ABPM coincided with the first night in only one sequence. A simple between-night comparison could therefore not separate ABPM from FNE [30]. We included both sequences in a single LMM to estimate the effects of ABPM and first-night status simultaneously, with ABPM sequence as a fixed effect [30,31]. A participant-specific random intercept accounted for the correlation between the two nightly measurements from each participant. The model was specified as follows:

*Yᵢⱼ* = *β*_0_ + *β_ABPM_* · *ABPM_ij_* + *β_FNE_* · *Night*1*ᵢⱼ* + *β_sequence_* · *Sequence_i_* + *b*_0_*_i_* + *ε_ij_*

In this model, *Yᵢⱼ* denotes the outcome for participant *i* on night *j*, and *β*_0_ denotes the intercept. *ABPM_ij_* was coded as 1 for a night with ABPM and 0 for a night without ABPM. *Night*1*ᵢⱼ* was coded as 1 for Night 1 and 0 for Night 2. *Sequence_i_* was coded as 1 for ABPM on Night 1 and 0 for ABPM on Night 2. The participant-specific random intercept *b*_0_*_i_* accounted for between-participant differences in baseline outcome levels, and *ε_ij_* denoted residual error. No interaction between ABPM and first-night status was included. Models were fitted using restricted maximum likelihood [32].

**Figure 2:**
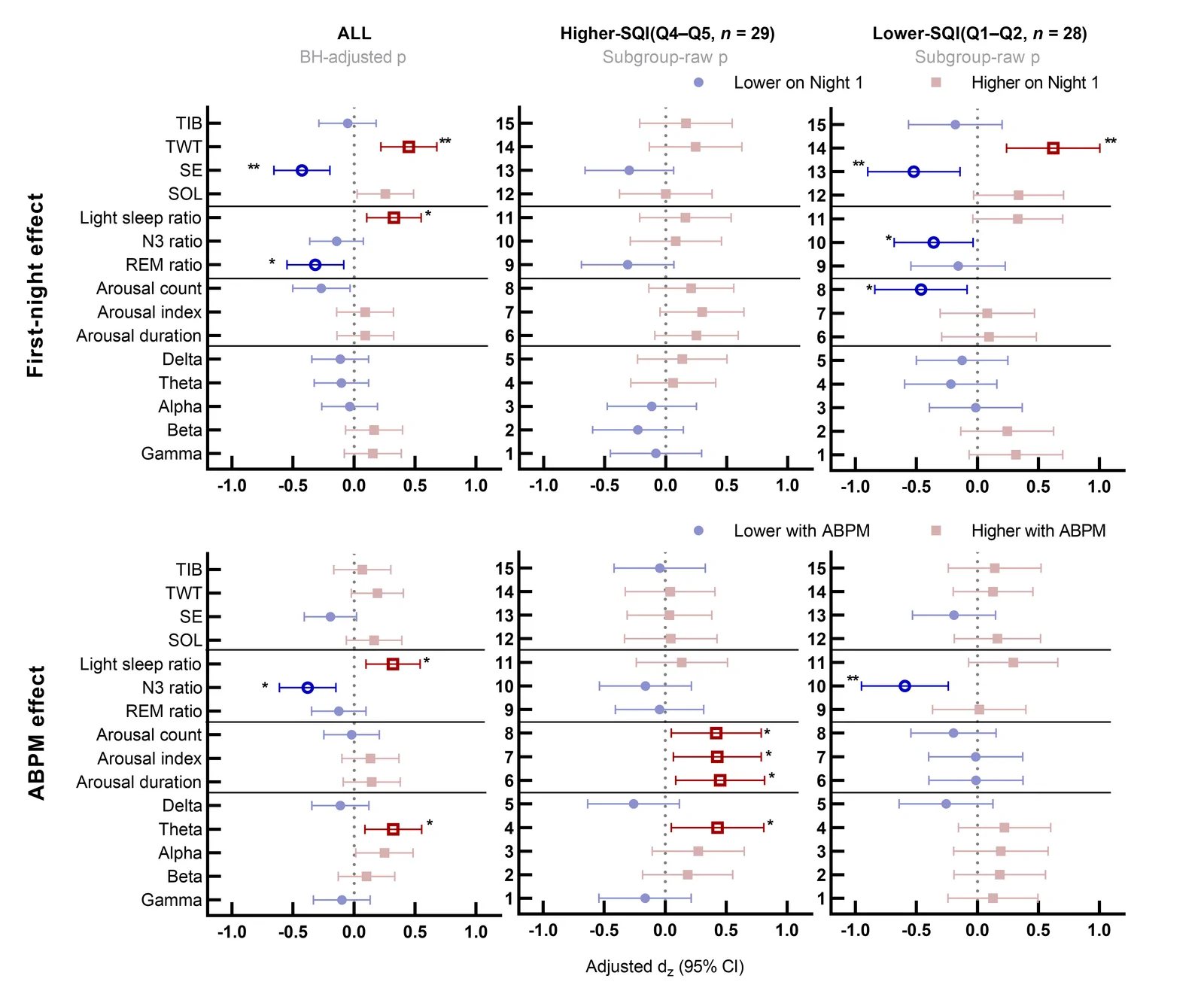
Effect-specific LMM estimates of FNE and ABPM in the full cohort (*n* = 72), higher-SQI group (Q4–Q5; *n* = 29), and lower-SQI group (Q1–Q2; *n* = 28). Points and error bars show adjusted standardized differences (*d_z_*) and pointwise 95% confidence intervals. FNE is Night 1 minus Night 2; ABPM is nights with ABPM minus nights without ABPM. Full-cohort significance symbols use BH-adjusted *p*-values from the 36 Table 2 tests. Darker symbols indicate statistically significant estimates under the p-value criterion specified for each panel. SQI-group analyses are exploratory and use unadjusted *p*-values. * *p* < 0.05, ** *p* < 0.01, using the stated *p*-value basis for each panel.

The LMM simultaneously estimated *β_ABPM_*, the adjusted mean difference between nights with and without ABPM, and *β_FNE_*, the adjusted mean difference between Night 1 and Night 2. Each coefficient was adjusted for the other night-level effect and ABPM sequence, while the participant-specific random intercept accounted for between-participant differences in baseline outcome levels. To compare effect sizes across outcomes, adjusted *d_z_* was calculated by dividing each coefficient by the standard deviation of the corresponding raw within-participant paired differences [33]. The resulting effect-specific estimates and their pointwise 95% confidence intervals are shown in Figure 2.

Estimated marginal means (EMMs) for the four conditions were derived from the same LMM by standardizing the ABPM sequence contribution while retaining the ABPM and FNEs. Overall differences among conditions were assessed with a joint F-test of the ABPM and FNE coefficients, with BH-FDR adjustment across outcomes. The six post hoc pairwise comparisons for each outcome used an approximate Tukey adjustment.

**Figure 3:**
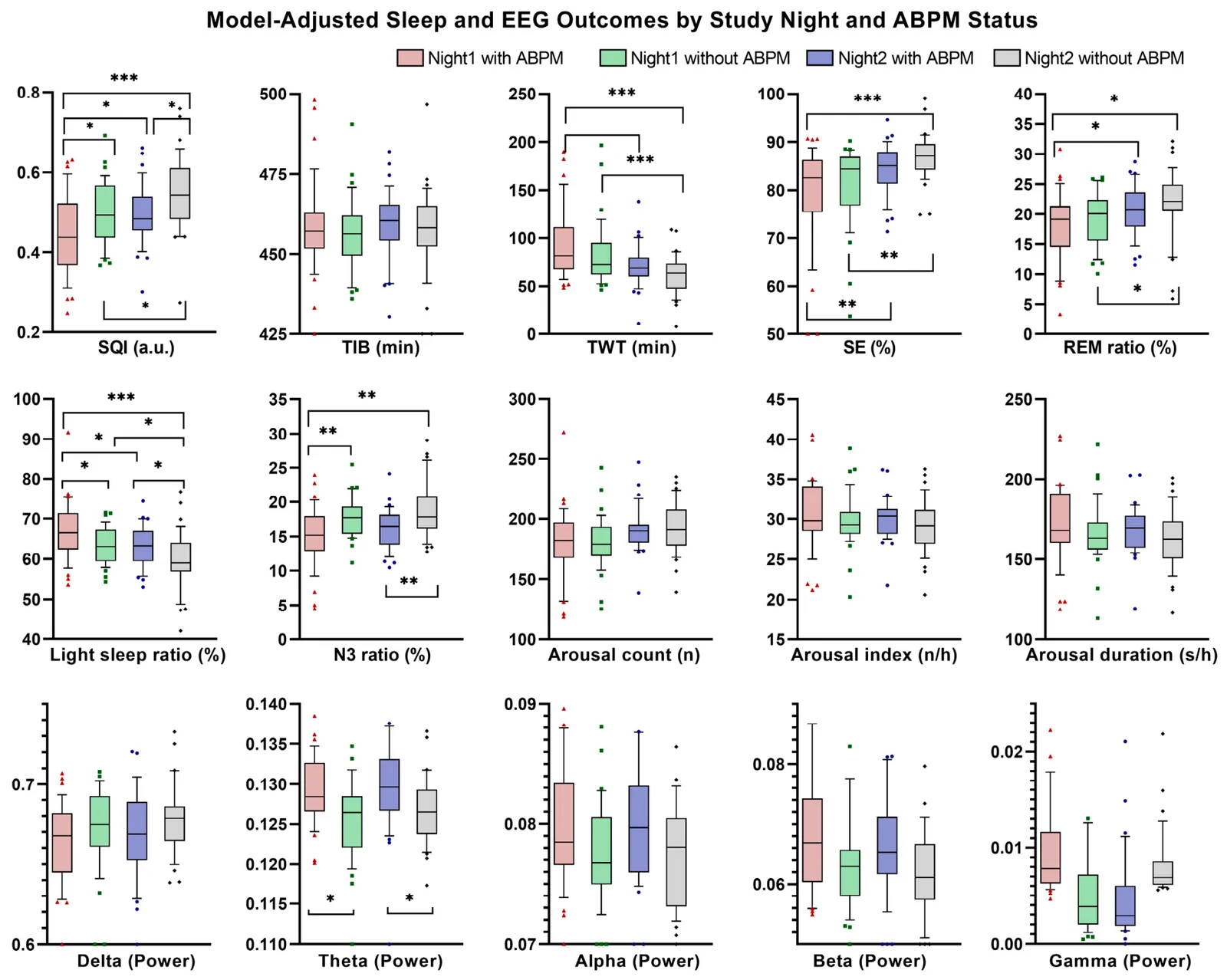
Model-adjusted sleep indices and relative EEG band power across the four combinations of study night and ABPM status. Each condition includes 36 observations; the 72 participants each contributed two conditions. Pink, green, purple, and gray indicate Night 1 with ABPM, Night 1 without ABPM, Night 2 with ABPM, and Night 2 without ABPM, respectively. Boxes show the median and interquartile range; whiskers show the 5th and 95th percentiles, and points mark observations outside the whiskers. Overall LMM tests were BH-FDR-adjusted across 18 outcomes. Brackets indicate significant pairwise comparisons after approximate Tukey adjustment, shown only for outcomes with overall BH-adjusted *p* < 0.05. * *p* < 0.05, ** *p* < 0.01, *** *p* < 0.001 (Tukey-adjusted pairwise comparisons).

Table 2 reports the LMM effect estimates, and Figure 3 presents model-adjusted condition summaries and pairwise comparisons. Participant-level adjusted values used in the displays were calculated with a partial-effects approach [34]. Supplementary Section S1 details the adjusted-value and EMM calculations and statistical comparisons. Supplementary Tables S1 and S2 provide observed and model-adjusted summaries, respectively.

### 2.4. Cuff-Related Analyses 

For each ABPM measurement, During ABPM was defined as a 2 min analysis window intended to encompass cuff inflation and deflation; Before ABPM was the preceding 30 s window. Measurements were excluded from the stable-sleep EEG analysis if the Before ABPM period contained wakefulness or an arousal detection. Participants with fewer than five eligible stable-sleep measurements were excluded from the cuff-related analyses.

#### 2.4.1. Cuff-Related EEG Power Analysis

A representative spectrogram was generated from EEG power estimated in 2 s analysis windows advanced in 0.25 s steps over the 0.5–35 Hz frequency range. For quantitative analysis, the EEG was band-pass filtered between 0.5 and 45 Hz. Power spectral density was then estimated separately for the Before ABPM and During ABPM periods using Welch’s method. To evaluate cuff-related EEG changes outside the first-night setting, the analysis was restricted to participants who underwent ABPM on Night 2. For each measurement and EEG frequency band, the median relative power was calculated separately for the Before ABPM and During ABPM periods. These measurement-level summaries were averaged within each participant, period, and frequency band. The resulting participant-level means were compared using two-sided paired *t*-tests with BH-FDR adjustment across the five frequency bands.

#### 2.4.2. Assessment of Sleep-Stage Transitions and Arousal Detections

Sleep-stage transitions and arousal detections were assessed for each ABPM measurement. Measurements with an arousal detection in the Before ABPM period were excluded, and the remaining measurements were grouped by their stage during that period. Within each baseline-stage group, we calculated the percentages showing a sleep-stage transition or containing at least one arousal detection during the During ABPM period. These percentages were descriptive, and repeated measurements from the same participant were counted separately.

### 2.5. Statistical Analysis

Continuous participant characteristics were summarized as mean ± standard deviation and categorical characteristics as *n* (%). BH-FDR adjustment was applied across the 36 ABPM and FNE coefficient tests in Table 2. Statistical tests were two-sided, with *p* < 0.05 considered significant after any applicable multiplicity adjustment.

Pearson correlations used two-night means for SQI and sleep indices and participant-level age; the methods and results are summarized in Supplementary Section S6. Analyses were performed in MATLAB R2025b (MathWorks, Natick, MA, USA) using the Statistics and Machine Learning Toolbox. Inputs for all 18 final night-level outcomes were complete across 144 participant-nights. This completeness does not rule out missing raw signals, preprocessing exclusions, or variable-specific missingness in Table 1.

## 3. Results

### 3.1. Participant Characteristics and Analysis Cohorts

The paired PSG dataset comprised 77 participants. Excluding five participants with fewer than 10 ABPM measurements left 72 participants contributing 144 participant-nights, with 36 in each randomized ABPM sequence (Table 1). These participants formed the cohort for the night-level LMM analyses and the source cohort for the effect-specific subgroup analyses. After the Section 2.4 criteria were applied, six participants had fewer than five stable-sleep measurements and were excluded, leaving 66 in the cuff-analysis source cohort.

### 3.2. Sleep Indices and EEG Changes Across Study Conditions

#### 3.2.1. SQI Correlations and Stratification

SQI correlated positively with sleep efficiency (r = 0.725) and N3-sleep percentage (r = 0.535), and negatively with wake after sleep onset (r = −0.716), age (r = −0.521), and arousal index (r = −0.332; Table S5). Correlations with SQI components partly reflected the construction of the score. SQI quintiles defined a lower-SQI group (Q1–Q2; *n* = 28), a middle group (Q3; *n* = 15), and a higher-SQI group (Q4–Q5; *n* = 29). Q3 participants were excluded only from SQI-stratified analyses.

Sensitivity analyses showed consistent negative ABPM and FNE associations across alternative SQI component sets, although effect magnitudes and statistical significance varied (Supplementary Section S5; Table S4 and Figure S1).

#### 3.2.2. Effect-Specific LMM Estimates

The LMM simultaneously estimated FNE- and ABPM-associated differences in the full cohort (Table 2). After BH-FDR adjustment across 36 tests, both FNE and ABPM remained associated with lower SQI (β = −0.049 and −0.051, respectively; both BH-adjusted *p* = 0.028). FNE was associated with shorter TST, longer TWT and WASO, lower SE, a higher light-sleep percentage, and a lower REM-sleep percentage. ABPM was associated with a higher light-sleep percentage, a lower N3-sleep percentage, and higher whole-night relative theta power.

#### 3.2.3. Exploratory LMM Analyses by SQI Group

Exploratory LMM analyses were conducted separately in the lower- and higher-SQI groups using unadjusted *p*-values (Figure 2). In the higher-SQI group, no FNE-associated result met the unadjusted significance threshold. In the lower-SQI group, FNE was nominally associated with higher TWT, lower SE, a lower N3-sleep percentage, and fewer arousals. ABPM was nominally associated with higher arousal count, arousal index, arousal duration per hour, and whole-night relative theta power in the higher-SQI group, and with a lower N3-sleep percentage in the lower-SQI group. Because the groups were analyzed separately, significance in one group and its absence in the other do not demonstrate a difference between groups.

#### 3.2.4. Four-Condition Analysis

Significant overall differences among the four conditions were observed for SQI, TWT, SE, light-sleep percentage, N3-sleep percentage, REM-sleep percentage, and relative theta power after BH-FDR adjustment (Figure 3). Model-adjusted summaries are provided in Supplementary Table S2.

### 3.3. Cuff-Related Findings

#### 3.3.1. Cuff-Related EEG Changes

**Figure 4:**
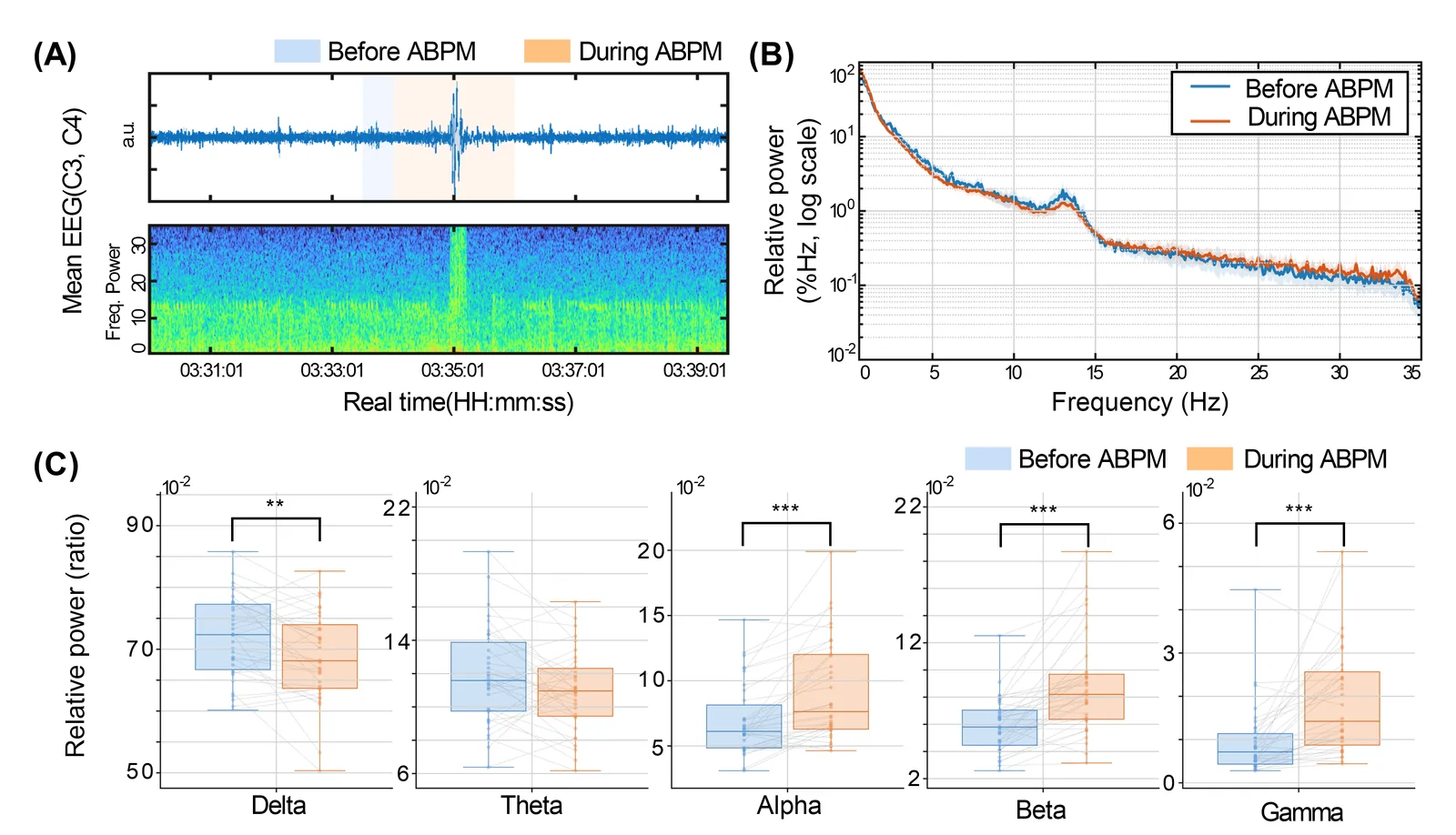
EEG activity during the Before ABPM and During ABPM periods on Night 2. The analysis included 34 participants and 322 stable-sleep measurements. (**A**) Representative EEG waveform and spectrogram. (**B**) Mean relative-power spectra on a logarithmic *y*-axis; shading indicates 95% CIs. (**C**) Participant-level relative power by frequency band; lines connect paired values. Blue and orange indicate Before ABPM and During ABPM, respectively. Each participant contributed one Before ABPM/During ABPM pair per EEG band after averaging measurement-level summaries within participants. Asterisks denote BH-adjusted *p*-values from two-sided participant-level paired *t*-tests across five EEG bands: ** *p* < 0.01, and *** *p* < 0.001.

The cuff-related EEG analysis used data from participants who underwent ABPM on Night 2. A representative waveform and spectrogram showed a brief change in EEG activity during an ABPM measurement (Figure 4A), and the relative power spectrum showed greater power at higher frequencies during ABPM (Figure 4B). Participant-level paired *t*-tests with BH-FDR adjustment across five bands identified significant differences in relative delta, alpha, beta, and gamma power, but no clear difference in theta power (Figure 4C).

#### 3.3.2. Sleep-Stage Transitions and Arousal Detections

**Figure 5:**
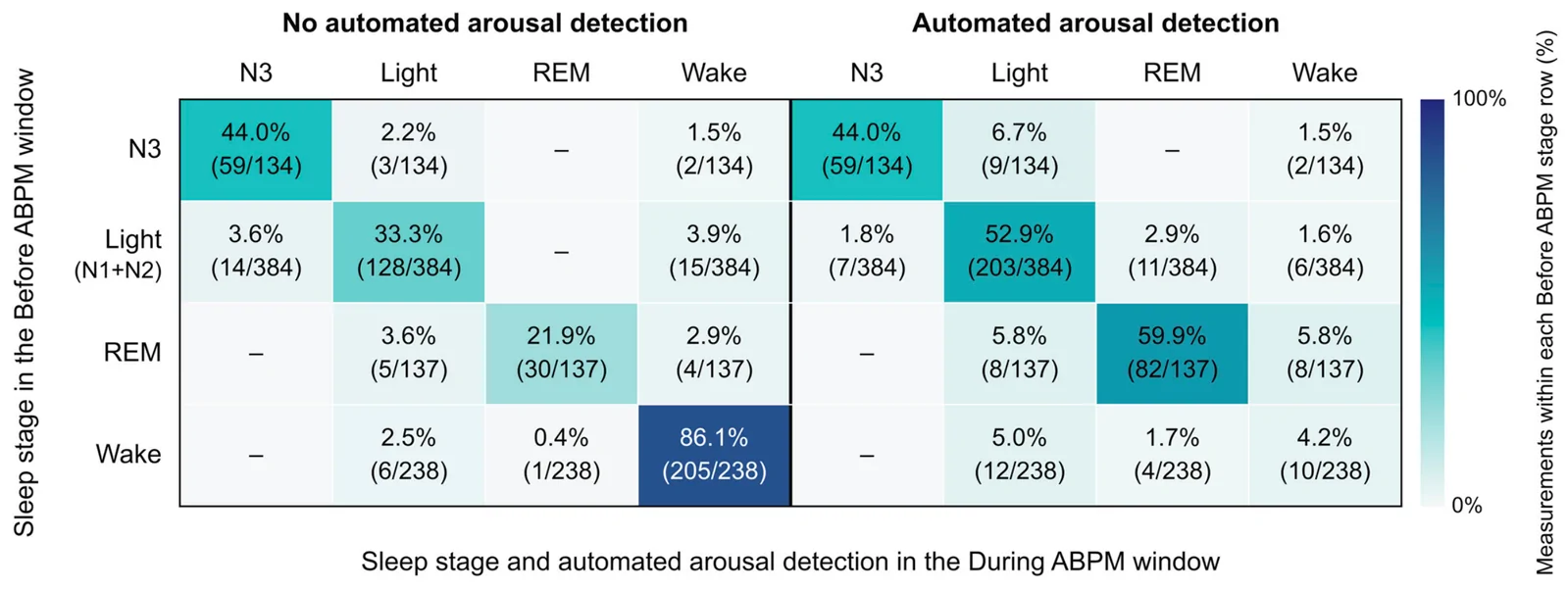
Sleep-stage transitions and automated arousal detections during ABPM. The matrix summarizes 893 eligible ABPM measurements from 66 participants. Rows show baseline sleep stage; columns show sleep stage and detection status in the During ABPM window. Detection-positive status denotes at least one positive detection within that window. Cells report measurement counts and percentages within each row. N1 and N2 were combined as light sleep.

After measurements with an arousal detection in the Before ABPM window were excluded, 893 ABPM measurements from the 66-participant cuff-analysis source cohort remained. Sleep-stage transitions and arousal detections were summarized as counts and percentages (Figure 5). In every baseline-stage group, more than 80% of measurements retained the same dominant sleep stage (range, 81.8–90.3%). Detection-positive proportions were highest for measurements beginning in REM sleep, followed by light sleep and N3 sleep. Of the 655 eligible measurements beginning during sleep, 395 (60.3%) contained at least one arousal detection within the 2 min During ABPM window. Most detection-positive measurements beginning during sleep retained the same dominant stage. Supplementary Table S3 provides detailed counts.

## 4. Discussion

### 4.1. Effects of FNE and ABPM on Sleep and EEG Activity

This two-night PSG study estimated ABPM- and FNE-associated differences simultaneously. Both were associated with altered sleep architecture and lower SQI, although their patterns differed. FNE was primarily associated with sleep duration and continuity, as well as a higher light-sleep percentage and a lower REM-sleep percentage, consistent with previous descriptions [1,2,3]. ABPM was associated with higher whole-night relative theta power after BH-FDR adjustment. Among outcomes with significant four-condition contrasts, sleep indices generally indicated poorer sleep quality on Night 1 with ABPM, when FNE and ABPM occurred together.

Analyses of individual cuff measurements provided complementary information. Among participants who underwent ABPM on Night 2, relative delta power was lower and relative alpha, beta, and gamma power were higher during ABPM than before ABPM. Of the 655 eligible measurements beginning during sleep, 395 (60.3%) contained at least one arousal detection within the 2 min During ABPM window. This pooled proportion describes temporal co-occurrence and does not estimate arousal attributable to cuff inflation.

The counterbalanced, within-participant design allowed us to estimate ABPM and FNE associations jointly while accounting for ABPM sequence and differences between participants. Combining whole-night analyses with analyses of individual measurements captured both changes across the night and transient EEG and arousal responses around ABPM measurements. Earlier studies often examined immediate responses to cuff inflation in smaller samples without jointly estimating ABPM and FNEs [4,6].

### 4.2. Interpretation of Arousal Findings

Previous PSG studies have reported EEG arousal or awakening during nocturnal cuff inflation [4,6]. In our study, detections coincided with ABPM measurements, but spontaneous events could not be separated from cuff-attributable arousals.

Arousal detections were present in 10.9% of measurements classified as wakefulness before ABPM (Figure 5). The detector’s handling of preceding sleep and transitions to wakefulness has not been validated against independent expert scoring. These observations should therefore not be equated with conventional sleep arousals [35,36].

Detection-positive proportions were 71.5% for measurements beginning in REM sleep, 59.1% in light sleep, and 52.2% in N3 sleep (Figure 5). These descriptive proportions cannot establish whether sleep stages differ in susceptibility to cuff inflation, given the uncertainty about detection accuracy across stages.

### 4.3. Limitations

An adaptation PSG night followed by randomized PSG nights with and without ABPM would provide a stronger design. The present crossover included a night without ABPM for each participant but no separate adaptation night. FNE was therefore estimated statistically rather than removed by design. We did not evaluate the benefits of an adaptation night or cuffless monitoring.

Multiplicity adjustments were applied within specified test families, but false-positive findings remain possible across the broader analysis, particularly in exploratory analyses using unadjusted *p*-values. Inference across the four conditions also depended on the additive LMM and an approximate Tukey adjustment for correlated model-estimated means (Supplementary Section S1).

The supplied sleep-stage annotations were not rescored according to AASM Version 3 (2023), so we could not quantify the impact of differences in scoring standards. The automated arousal detector was not validated against independent blinded expert scoring. Its accuracy and the consistency of detection errors across conditions and sleep stages therefore remain uncertain. Such errors could affect arousal-related outcomes, the arousal-index component of SQI, and measurement eligibility.

Measurements with baseline arousal detections were excluded, and no duration- and stage-matched windows without ABPM were included. These features prevented us from separating spontaneous arousals from those attributable to cuff inflation; the detection proportions therefore describe temporal co-occurrence.

Findings from nominally healthy adults may not generalize to patients with hypertension or cardiovascular disease, whose sleep architecture and arousal characteristics may differ [37,38,39,40]. The type and quantification of arousal also matter when interpreting cardiovascular relevance [41]. The proportion of measurement windows containing arousal detections differs from the cumulative arousal duration evaluated in long-term cohort research [42]. Its prognostic significance cannot be inferred from the present results.

SQI is a research composite based on ranks within the study sample. Its quintile boundaries and changes in score have no established clinical interpretation or thresholds and should not be transferred directly to other cohorts. Correlation and component-selection analyses characterize the score within this dataset; its reliability, external validity, and clinical interpretation require independent validation.

## 5. Conclusions

In this secondary analysis of a counterbalanced two-night PSG crossover, FNE and ABPM were associated with poorer sleep quality, reflected by lower SQI scores. FNE was associated with changes in sleep continuity and architecture, whereas ABPM was associated with a higher light-sleep percentage, a lower N3-sleep percentage, and higher whole-night relative theta power. Individual ABPM measurements coincided with lower relative delta power and higher relative alpha, beta, and gamma power. Arousal detections were also observed within the measurement windows. These observations describe temporal co-occurrence and do not, by themselves, establish that cuff inflation caused arousal. An adaptation PSG night may be considered when feasible. Cuffless blood pressure monitoring may also be considered once its accuracy has been established for the intended setting. Applicability to patients with hypertension or cardiovascular disease requires separate evaluation.

## Figures and Tables

**Table 1:** Participant characteristics for the full cohort and by randomized ABPM sequence. Values are presented as mean ± standard deviation or *n* (%). SBP and DBP were measured at baseline.

Characteristic	Unit	Overall (*n* = 72)	ABPM on Night 1 (*n* = 36)	ABPM on Night 2 (*n* = 36)
Participants	*n*	72	36	36
Age	years	34.24 ± 14.58	36.53 ± 15.59	31.94 ± 13.33
Male	*n* (%)	38 (52.78%)	20 (55.56%)	18 (50.00%)
Height	cm	165.62 ± 9.49	165.92 ± 10.40	165.33 ± 8.62
Weight	kg	63.94 ± 12.00	64.52 ± 13.01	63.35 ± 11.05
BMI	kg/m^2^	23.21 ± 3.17	23.25 ± 2.65	23.17 ± 3.66
Waist circumference	cm	77.19 ± 9.45	76.89 ± 8.81	77.48 ± 10.16
SBP	mmHg	113.34 ± 16.22	115.19 ± 16.17	111.49 ± 16.28
DBP	mmHg	72.41 ± 8.42	72.42 ± 7.18	72.40 ± 9.61

BMI, body mass index; ABPM, ambulatory blood pressure monitoring; SBP, systolic blood pressure; DBP, diastolic blood pressure.

**Table 2:** Adjusted FNE and ABPM estimates from linear mixed-effects models in 72 participants. The FNE coefficient (β) represents Night 1 minus Night 2, and the ABPM coefficient represents nights with ABPM minus nights without ABPM. Adjusted *d_z_* values are standardized paired differences. Confidence intervals are pointwise 95% intervals; *p*-values are BH-FDR-adjusted across the 36 ABPM and FNE tests. * *p* < 0.05, ** *p* < 0.01.

Outcome	Unit	FNE β [95% CI]	FNE dz [95% CI]	FNE *p* (BH-FDR)	ABPM β [95% CI]	ABPM dz [95% CI]	ABPM *p* (BH-FDR)
SQI	score	−0.049 [−0.085, −0.013]	−0.305 [−0.527, −0.082]	0.0281 *	−0.051 [−0.087, −0.015]	−0.316 [−0.539, −0.093]	0.0281 *
Time in bed (TIB)	min	−1.31 [−7.02, 4.41]	−0.054 [−0.288, 0.181]	0.6903	1.64 [−4.07, 7.35]	0.067 [−0.167, 0.302]	0.6233
Total sleep time (TST)	min	−25.41 [−40.73, −10.09]	−0.385 [−0.617, −0.153]	0.0135 *	−9.74 [−25.06, 5.58]	−0.139 [−0.357, 0.080]	0.3476
Total wake time (TWT)	min	22.21 [10.86, 33.56]	0.449 [0.220, 0.679]	0.0060 **	10.21 [−1.14, 21.56]	0.192 [−0.021, 0.405]	0.1745
Sleep efficiency (SE)	%	−5.56 [−8.55, −2.57]	−0.427 [−0.656, −0.197]	0.0061 **	−2.70 [−5.69, 0.29]	−0.194 [−0.409, 0.021]	0.1745
Sleep-onset latency (SOL)	min	7.98 [0.79, 15.17]	0.257 [0.025, 0.488]	0.0829	5.19 [−2.01, 12.38]	0.164 [−0.063, 0.391]	0.3307
Wake after sleep onset (WASO)	min	12.37 [2.72, 22.02]	0.297 [0.065, 0.530]	0.0406 *	6.03 [−3.62, 15.69]	0.140 [−0.084, 0.365]	0.3476
Light sleep	% of TST	3.63 [1.16, 6.10]	0.327 [0.105, 0.549]	0.0281 *	3.55 [1.08, 6.02]	0.319 [0.097, 0.541]	0.0281 *
N3 sleep	% of TST	−0.99 [−2.49, 0.52]	−0.143 [−0.362, 0.076]	0.3476	−2.47 [−3.98, −0.96]	−0.380 [−0.612, −0.148]	0.0135 *
REM sleep	% of TST	−2.64 [−4.58, −0.71]	−0.318 [−0.550, −0.085]	0.0281 *	−1.08 [−3.02, 0.85]	−0.125 [−0.348, 0.098]	0.3899
Arousal count	count	−11.19 [−20.97, −1.42]	−0.269 [−0.503, −0.034]	0.0752	−0.86 [−10.63, 8.91]	−0.020 [−0.246, 0.207]	0.8619
Arousal index	/hour	0.57 [−0.88, 2.02]	0.091 [−0.142, 0.324]	0.4953	0.83 [−0.62, 2.28]	0.134 [−0.100, 0.368]	0.3881
Arousal duration per hour	s/h	3.25 [−4.96, 11.47]	0.092 [−0.140, 0.324]	0.4953	5.10 [−3.12, 13.31]	0.145 [−0.089, 0.379]	0.3476
Relative delta power	power	−0.00697 [−0.02129, 0.00736]	−0.11335 [−0.34649, 0.11978]	0.4531	−0.00694 [−0.02127, 0.00739]	−0.11294 [−0.34607, 0.12018]	0.4531
Relative theta power	power	−0.00129 [−0.00403, 0.00146]	−0.10445 [−0.32759, 0.11870]	0.4582	0.00378 [0.00103, 0.00652]	0.32110 [0.08787, 0.55434]	0.0281 *
Relative alpha power	power	−0.00041 [−0.00302, 0.00220]	−0.03595 [−0.26357, 0.19167]	0.7769	0.00277 [0.00016, 0.00538]	0.24858 [0.01409, 0.48307]	0.0975
Relative beta power	power	0.00404 [−0.00170, 0.00977]	0.16423 [−0.06918, 0.39763]	0.3328	0.00252 [−0.00322, 0.00825]	0.10150 [−0.12997, 0.33298]	0.4709
Relative gamma power	power	0.00566 [−0.00295, 0.01427]	0.15340 [−0.08003, 0.38683]	0.3476	−0.00374 [−0.01235, 0.00487]	−0.10061 [−0.33249, 0.13126]	0.4709

ABPM, ambulatory blood pressure monitoring; CI, confidence interval; FNE, first-night effect; SQI, sleep quality index; d_z_, adjusted standardized paired difference.

## Data Availability

The data analyzed in this study were originally collected and published byYilmaz et al. [10] and obtained for this analysis under a data access agreement with the National University of Singapore (NUS). They are not publicly available because of institutional restrictions. Requests for access should be directed to NUS and are subject to institutional approval and the terms of the data access agreement.

## Supplementary Materials

The following supporting information can be downloaded at: https://www.mdpi.com/article/10.3390/jcm15218759/s1. Supplementary Section S1 describes the calculation of model-adjusted values using a partial-effects approach [34]; Section S2–S7; Tables S1–S6 and Figure S1.
