# Supplementary material for: Effects of Ambulatory Blood Pressure Monitoring During Polysomnography on Sleep Indices and EEG Power
Source: J Clin Med. 2026 Nov 9;15(21):8759. doi: 10.3390/jcm15218759 (PMC13763799; doi:10.3390/jcm15218759)
Supplement: Supplementary file 1 [file jcm-15-08759-s001.zip › jcm-4622900-supplementary.pdf]

Supplementary Materials

This file presents observed night-level summaries and model-adjusted values for the four study conditions, together with analysis denominators and measurement-level counts. It also describes SQI component-selection sensitivity analyses, correlations of SQI with age and PSG-derived sleep indices, and implementation of the study-specific automated arousal detector. Tables present summary values rather than separate rows for individual participants.

Section S1. Calculation of Model-Adjusted Outcome Values

Adjusted values were calculated from the fitted linear mixed-effects model using a partial-effects approach [34]. A and F denote the night-level ABPM and Night 1 indicators, respectively, and S denotes the participant-level sequence indicator defined in Section 2.3. For participant i on night j, the conditional residual was calculated by removing the fitted fixed effects and participant-specific random intercept:

$$\varepsilon_{ij}^{cond} = Y_{ij} - \left(\hat{\beta}_0 + \hat{\beta}_{ABPM}A_{ij} + \hat{\beta}_{FNE}F_{ij} + \hat{\beta}_{sequence}S_i + \hat{b}_{0i}\right)$$

The adjusted value was then calculated by setting the sequence contribution to a common reference value of 0.5:

$$\mathrm{Y}_{\mathrm{ij}}^{\mathrm{adj}} = \hat{\beta}_0 + \hat{\beta}_{ABPM}A_{ij} + \hat{\beta}_{FNE}F_{ij} + 0.5\hat{\beta}_{sequence} + \varepsilon_{ij}^{cond}$$

Equivalently, the adjusted value can be written directly from the observed outcome:

$$\mathrm{Y}_{\mathrm{ij}}^{\mathrm{adj}} = \mathrm{Y}_{ij} - \hat{\beta}_{sequence}(S_i - 0.5) - \hat{b}_{0i}$$

The adjusted values retain the ABPM and FNE contributions while accounting for sequence and participant-specific baseline differences. In effect-specific comparisons, the effect of interest is retained while the other night-level effect is also accounted for. Participant-level adjusted values were calculated using the partial-effects equations above. Statistical comparisons among the four conditions used the fixed-effect estimates and coefficient covariance from the same LMM as Table 2. Estimated marginal means (EMMs) were evaluated at a common sequence reference of 0.5. Joint $F(2,140)$ tests of the ABPM and FNE coefficients were BH-FDR-adjusted across 18 outcomes. The six pairwise condition contrasts for each outcome used an approximate studentized-range Tukey adjustment. Brackets in Figure 3 required both a joint-test BH-adjusted $p < 0.05$ and a pairwise Tukey-adjusted $p < 0.05$. Effect-specific estimates in Figure 2 were obtained from the corresponding LMM outputs. Full-cohort symbols used the BH-adjusted p values from Table 2, whereas exploratory subgroup symbols used unadjusted p values.

The coefficient covariance matrix used for the four-condition joint tests and contrasts was reconstructed from the standard errors associated with the Table 2 LMM under the complete, balanced two-night random-intercept design. The covariance reconstruction was checked against raw-data GLS calculations, closed-form REML variance estimates, and EMM output. The joint tests and pairwise contrasts used the Table 2 LMM estimates with 140 denominator degrees of freedom. Tukey probabilities were calculated using $q = \sqrt{2}|t|$ with four means and 140 degrees of freedom. This provided an approximation rather than an exact joint-distribution adjustment for the correlated EMMs.

Section S2. Observed Night-Level Values

Observed night-level values are presented as mean ± SD without LMM adjustment. They summarize the 72 participants with paired two-night PSG and at least 10 ABPM measurements, comprising 144 participant-nights and 36 observations per condition. Each participant contributed two of the four combinations of study night and ABPM status.

Table S1. Observed night-level sleep indices and relative EEG band power across the four study conditions.

Outcome	Unit	N1/ABPM+ (n=36)	N1/ABPM− (n=36)	N2/ABPM+ (n=36)	N2/ABPM− (n=36)
SQI	score	0.445 ± 0.167	0.500 ± 0.160	0.498 ± 0.154	0.544 ± 0.157
Time in bed	min	456.92 ± 53.91	456.94 ± 55.47	459.89 ± 52.98	456.58 ± 48.49
Total sleep time	min	359.65 ± 87.88	372.36 ± 59.81	388.03 ± 44.92	394.81 ± 48.38
Total wake time	min	94.19 ± 55.65	82.78 ± 57.23	70.78 ± 41.47	61.78 ± 25.07
Sleep efficiency	%	78.24 ± 15.64	81.88 ± 11.11	84.74 ± 7.72	86.50 ± 5.27
Sleep-onset latency	min	45.76 ± 38.20	34.60 ± 17.80	31.81 ± 14.17	32.60 ± 12.02
Wake after sleep onset	min	44.47 ± 42.20	41.89 ± 43.11	35.56 ± 33.62	26.07 ± 20.77
Light sleep	% of TST	67.49 ± 11.65	62.54 ± 9.51	62.46 ± 10.28	60.30 ± 11.16
N3 sleep	% of TST	15.03 ± 8.81	17.78 ± 7.25	16.30 ± 7.20	18.48 ± 7.88
REM sleep	% of TST	17.49 ± 7.97	19.68 ± 6.56	21.24 ± 6.28	21.22 ± 7.38
Arousal count	count	188.28 ± 69.70	172.81 ± 44.53	183.14 ± 36.42	200.33 ± 52.80
Arousal index	/hour	32.07 ± 10.00	28.19 ± 6.88	28.45 ± 5.28	30.67 ± 7.99
Arousal duration per hour	s/h	179.71 ± 57.26	157.70 ± 39.34	159.54 ± 30.00	171.36 ± 45.12
Relative delta power	power	0.65153 ± 0.07856	0.68199 ± 0.08189	0.68202 ± 0.06586	0.66544 ± 0.07008
Relative theta power	power	0.13374 ± 0.02237	0.12052 ± 0.02675	0.12559 ± 0.02793	0.13125 ± 0.02192
Relative alpha power	power	0.08594 ± 0.03188	0.07099 ± 0.02185	0.07417 ± 0.02535	0.08359 ± 0.03048
Relative beta power	power	0.06914 ± 0.03422	0.06562 ± 0.03428	0.06410 ± 0.02006	0.06258 ± 0.03278
Relative gamma power	power	0.00720 ± 0.00641	0.01664 ± 0.05320	0.00725 ± 0.00490	0.00528 ± 0.00382

Values are unadjusted observed summaries. The values in Table S2 should be used when reproducing the adjusted four-condition display in Figure 3.

Section S3. Model-Adjusted Four-Condition Values for Figure 3

Values are presented as model-adjusted mean ± SD of participant-level adjusted values [pointwise 95% CI]. Each condition contains 36 observations from the 72 participants, each of whom contributed observations to two conditions. The condition labels are N1/ABPM+ (Night 1 with ABPM), N1/ABPM− (Night 1 without ABPM), N2/ABPM+ (Night 2 with ABPM), and N2/ABPM− (Night 2 without ABPM).

Table S2. Model-adjusted four-condition outcome values for Figure 3.

Outcome	Unit	N1/ABPM+ (n=36)	N1/ABPM− (n=36)	N2/ABPM+ (n=36)	N2/ABPM− (n=36)
SQI	score	0.447 ± 0.100 [0.406, 0.488]	0.498 ± 0.078 [0.456, 0.539]	0.496 ± 0.074 [0.455, 0.537]	0.547 ± 0.096 [0.505, 0.588]
Time in bed	min	457.75 ± 14.61 [445.13, 470.37]	456.11 ± 11.30 [443.49, 468.73]	459.06 ± 10.59 [446.43, 471.68]	457.42 ± 13.48 [444.79, 470.04]
Total sleep time	min	361.14 ± 55.24 [344.67, 377.60]	370.88 ± 35.47 [354.41, 387.35]	386.55 ± 25.76 [370.08, 403.01]	396.29 ± 31.60 [379.82, 412.76]
Total wake time	min	93.59 ± 36.49 [81.32, 105.86]	83.38 ± 32.42 [71.11, 95.65]	71.38 ± 21.66 [59.11, 83.65]	61.17 ± 20.22 [48.90, 73.44]
Sleep efficiency	%	78.71 ± 12.05 [75.81, 81.61]	81.41 ± 7.95 [78.51, 84.31]	84.27 ± 5.20 [81.37, 87.17]	86.97 ± 4.74 [84.07, 89.87]
Sleep-onset latency	min	42.77 ± 34.72 [36.31, 49.23]	37.59 ± 15.59 [31.13, 44.05]	34.80 ± 12.18 [28.34, 41.26]	29.61 ± 11.55 [23.15, 36.07]
Wake after sleep onset	min	46.20 ± 30.70 [36.51, 55.89]	40.16 ± 29.15 [30.47, 49.85]	33.83 ± 22.16 [24.14, 43.52]	27.80 ± 16.67 [18.11, 37.48]
Light sleep	% of TST	66.79 ± 7.38 [64.01, 69.57]	63.24 ± 4.39 [60.46, 66.02]	63.16 ± 4.93 [60.38, 65.94]	59.61 ± 7.13 [56.83, 62.38]
N3 sleep	% of TST	15.17 ± 4.33 [13.20, 17.14]	17.64 ± 2.85 [15.67, 19.61]	16.16 ± 2.83 [14.19, 18.13]	18.63 ± 3.95 [16.66, 20.59]
REM sleep	% of TST	18.04 ± 5.98 [16.13, 19.95]	19.12 ± 4.49 [17.21, 21.04]	20.69 ± 4.28 [18.77, 22.60]	21.77 ± 5.58 [19.86, 23.68]
Arousal count	count	180.11 ± 29.09 [166.98, 193.25]	180.97 ± 21.49 [167.84, 194.11]	191.31 ± 18.34 [178.17, 204.44]	192.17 ± 21.22 [179.03, 205.30]
Arousal index	/hour	30.54 ± 4.22 [28.60, 32.49]	29.71 ± 3.26 [27.77, 31.65]	29.98 ± 2.62 [28.04, 31.92]	29.15 ± 3.29 [27.20, 31.09]
Arousal duration per hour	s/h	171.25 ± 23.92 [160.19, 182.30]	166.15 ± 18.66 [155.10, 177.21]	168.00 ± 14.99 [156.94, 179.05]	162.90 ± 18.31 [151.84, 173.95]
Relative delta power	power	0.663 ± 0.027 [0.645, 0.682]	0.670 ± 0.044 [0.651, 0.689]	0.670 ± 0.038 [0.652, 0.689]	0.677 ± 0.022 [0.658, 0.696]
Relative theta power	power	0.129 ± 0.004 [0.123, 0.135]	0.125 ± 0.007 [0.119, 0.131]	0.130 ± 0.008 [0.124, 0.136]	0.127 ± 0.004 [0.121, 0.132]
Relative alpha power	power	0.080 ± 0.006 [0.073, 0.086]	0.077 ± 0.006 [0.070, 0.084]	0.080 ± 0.006 [0.074, 0.087]	0.077 ± 0.005 [0.071, 0.084]
Relative beta power	power	0.069 ± 0.011 [0.061, 0.076]	0.066 ± 0.018 [0.058, 0.074]	0.065 ± 0.013 [0.057, 0.072]	0.062 ± 0.010 [0.054, 0.070]
Relative gamma power	power	0.010 ± 0.006 [0.002, 0.018]	0.014 ± 0.050 [0.006, 0.021]	0.004 ± 0.005 [-0.003, 0.012]	0.008 ± 0.003 [0.001, 0.016]

CI, confidence interval; SD, standard deviation of participant-level adjusted values. EEG entries represent relative band power. SD describes the spread of the participant-level model-adjusted values, whereas the pointwise 95% CI is the model-based interval for the estimated marginal mean. These intervals are neither simultaneous confidence intervals nor Tukey-adjusted pairwise confidence intervals.

Table S1 presents observed summaries for the four study conditions. Table S2 presents model-adjusted summaries corresponding to the distributions shown in Figure 3. Participant-level adjusted values were calculated using the partial-effects approach described in Appendix S1 and Section 2.3 of the main manuscript.

Section S4. Measurement-Level Sleep-Stage Transitions and Arousal Detections

The measurement-level analysis included 893 eligible ABPM measurements from 66 participants after measurements with an arousal detection in the Before ABPM window were excluded. Percentages were calculated within each baseline-stage group. Detection-positive status denotes at least one arousal detection within the 2-min During ABPM window. Repeated measurements from the same participant were counted separately. This descriptive analysis does not estimate arousal attributable to cuff inflation.

Of the 655 eligible ABPM measurements beginning during sleep, 395 (60.3%) contained at least one arousal detection within the 2-min During ABPM window. This pooled measurement-level proportion describes temporal co-occurrence and does not estimate arousal attributable to cuff inflation. It was calculated across measurements rather than as a mean of participant-specific proportions. The Before ABPM and During ABPM windows differed in duration (30 s and 2 min), and no comparison was made with non-measurement windows matched for duration and sleep stage.

Table S3. Sleep-stage transitions and automated arousal detections during individual ABPM measurements.

Before ABPM stage	Eligible measurements	Arousal detection	Same dominant stage during ABPM
N3	134	70/134 (52.2%)	118/134 (88.1%)
Light sleep (N1+N2)	384	227/384 (59.1%)	331/384 (86.2%)
REM	137	98/137 (71.5%)	112/137 (81.8%)
Wake	238	26/238 (10.9%)	215/238 (90.3%)

ABPM, ambulatory blood pressure monitoring; REM, rapid eye movement. The same-stage percentage is calculated irrespective of arousal detection status. A measurement can therefore retain the same dominant stage while containing at least one arousal detection. Measurements beginning in wakefulness are reported separately and excluded from the 395/655 sleep-start detection proportion.

Section S5. SQI Component-Selection Sensitivity Analysis

We randomly sampled 1,000 alternative SQI component sets while retaining the scoring directions, rank transformation, and equal weighting described in Section 2.2.2. The original VIF threshold and domain balance were not required for these alternative sets.

The analyses included 72 participants and used a common ranking reference of 144 participant-nights. VIFs and Pearson correlations were calculated using participant-level two-night means, with age treated as a participant-level variable. ABPM and FNE coefficients were estimated by regressing within-participant SQI differences on sequence (two-sided unadjusted tests; residual df = 70).

Table S4 summarizes coefficients by maximum VIF, and Figure S1 shows correlation profiles. Because the VIF groups differed in composition and size, these comparisons do not identify an optimal VIF cutoff.

Table S4. ABPM and FNE coefficients by maximum VIF across 1,000 alternative SQI component sets.

Maximum VIF	SQI component sets, n	ABPM coefficient Median [minimum, maximum]	ABPM: Significant decrease, n (%)	FNE coefficient Median [minimum, maximum]	FNE: Significant decrease, n (%)
≤10	8	−0.0565 [−0.0596, −0.0516]	8 (100.0%)	−0.0536 [−0.0652, −0.0402]	8 (100.0%)
10<VIF≤30	60	−0.0575 [−0.0688, −0.0494]	60 (100.0%)	−0.0575 [−0.0757, −0.0376]	60 (100.0%)
30<VIF≤100	380	−0.0533 [−0.0710, −0.0414]	380 (100.0%)	−0.0513 [−0.0825, −0.0302]	349 (91.8%)
100<VIF≤300	35	−0.0602 [−0.0686, −0.0515]	35 (100.0%)	−0.0488 [−0.0725, −0.0283]	25 (71.4%)
300<VIF≤1,000	459	−0.0484 [−0.0675, −0.0345]	456 (99.3%)	−0.0383 [−0.0684, −0.0138]	261 (56.9%)
1,000<VIF ≤10,000	2	−0.0446 [−0.0446, −0.0446]	2 (100.0%)	−0.0276 [−0.0284, −0.0268]	0 (0.0%)
VIF>10,000	56	−0.0406 [−0.0495, −0.0306]	51 (91.1%)	−0.0250 [−0.0402, −0.0095]	2 (3.6%)
All	1,000	−0.0511 [−0.0710, −0.0306]	992 (99.2%)	−0.0445 [−0.0825, −0.0095]	705 (70.5%)

n denotes the number of component sets. Coefficients are reported as medians [minimum, maximum], not confidence intervals. Contrasts are ABPM minus non-ABPM and Night 1 minus Night 2. Significant decreases were defined by two-sided unadjusted $p < 0.05$, separately from the primary BH-FDR analyses.

These analyses assess sensitivity within this dataset; they do not evaluate independent validity or reliability.

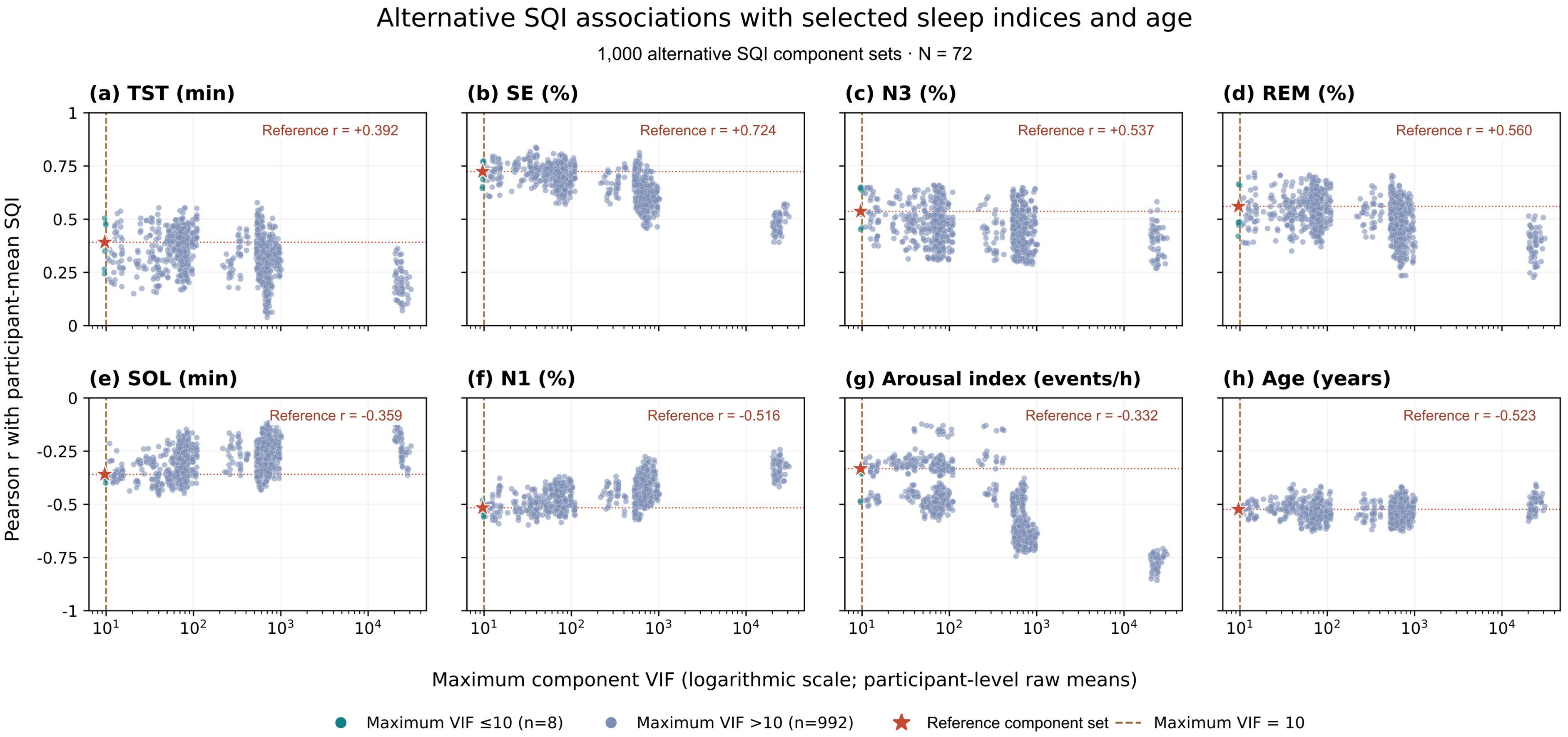

Figure S1. Correlations of alternative SQIs with (a) TST, (b) SE, (c) N3, (d) REM, (e) SOL, (f) N1, (g) arousal index, and (h) age. Each panel shows results for 1,000 component sets. The axes show maximum VIF on a logarithmic scale and Pearson r, calculated using two-night means for SQI and sleep indices and participant-level age. Colors distinguish sets with maximum VIF ≤ 10 (n = 8) from those with maximum VIF >10 (n = 992). Stars mark the reference set scored using the same 144 participant-nights. Correlations with SQI components partly reflect score construction.

Section S6. Associations of SQI with Age and PSG-Derived Sleep Indices

Table S5 reports Pearson correlations between the manuscript SQI and age and 13 PSG-derived sleep indices. Correlations were calculated using two-night means for SQI and sleep indices and participant-level age (n = 72).

Table S5. Pearson correlations of SQI with age and the 13 PSG-derived sleep indices reported in the main manuscript.

Metric (unit)	In SQI (direction)	Participants, N	Pearson r	Pointwise 95% CI
Age (years)	No	72	-0.5208	[-0.671, -0.329]
Time in bed (min)	No	72	-0.1866	[-0.401, 0.047]
Total sleep time (min)	Yes (+)	72	0.3917	[0.176, 0.572]
Total wake time (min)	No	72	-0.7634	[-0.845, -0.646]
Sleep efficiency (%)	Yes (+)	72	0.7253	[0.593, 0.819]
Sleep onset latency (min)	Yes (−)	72	-0.3577	[-0.544, -0.137]
Wake after sleep onset (min)	No	72	-0.7156	[-0.813, -0.580]
N1 sleep (% of TST)	Yes (−)	72	-0.5195	[-0.670, -0.327]
N3 sleep (% of TST)	Yes (+)	72	0.5348	[0.346, 0.682]
REM sleep (% of TST)	Yes (+)	72	0.5613	[0.379, 0.702]
Light sleep, N1+N2 (% of TST)	No	72	-0.7563	[-0.841, -0.636]
Arousal count	No	72	-0.0593	[-0.287, 0.175]
Arousal index (events/h)	Yes (−)	72	-0.3324	[-0.524, -0.109]
Arousal duration per hour (s/h)	No	72	-0.3310	[-0.523, -0.108]

CIs are pointwise 95% Fisher-z intervals. Yes (+/−) identifies SQI components for which higher scores correspond to higher/lower raw values. Correlations with SQI components partly reflect score construction and do not establish independent validity.

Section S7. Study-Specific Automated Arousal Detector

Table S6 specifies the signal-processing parameters used for the detection criteria and event quantification described in Sections 2.1.3 and 2.2.1. Numerical signal thresholds were defined for this study, and the same settings were applied across participants and study conditions.

Table S6. Arousal detection criteria and event quantification.

Component	Criterion
EEG input	The C3–A2 and C4–A1 signals were averaged, then band-pass filtered at 0.3–64 Hz.
Candidate detection	Power in the theta (4–7 Hz), alpha (8–13 Hz), and high-frequency (16–64 Hz) bands was estimated using a 1-s moving mean of the squared band-filtered signal. Candidate segments required power in at least one band to be ≥1.5 times its 10-s moving median.
Spindle exclusion	The Hilbert amplitude envelope of the 11–16 Hz EEG signal was divided by its 30-s moving median. Intervals with a ratio ≥2.0 lasting 0.5–2.0 s were treated as estimated spindles and removed from candidate segments.
Minimum duration	At least 3 s after spindle exclusion.
Preceding sleep	The preceding 10 s had to contain nonmissing sleep-stage labels, no wakefulness, and no accepted detection.
REM criterion	Chin EMG was filtered at 10–100 Hz, and its Hilbert amplitude envelope was smoothed over 0.2 s. For candidates whose modal sleep stage was REM, elevation to ≥2 times the preceding 10-s baseline median had to persist for ≥1 continuous second overlapping both the candidate and REM labels.
Event quantification	Each 2-s interval containing an accepted detection was marked positive, and consecutive positive intervals were grouped into an event. Only events beginning during sleep were counted. Event duration was the number of positive intervals × 2 s.
